# The WNT1^*G177C*^ mutation specifically affects skeletal integrity in a mouse model of osteogenesis imperfecta type XV

**DOI:** 10.1038/s41413-021-00170-0

**Published:** 2021-11-10

**Authors:** Nele Vollersen, Wenbo Zhao, Tim Rolvien, Fabiola Lange, Felix Nikolai Schmidt, Stephan Sonntag, Doron Shmerling, Simon von Kroge, Kilian Elia Stockhausen, Ahmed Sharaf, Michaela Schweizer, Meliha Karsak, Björn Busse, Ernesto Bockamp, Oliver Semler, Michael Amling, Ralf Oheim, Thorsten Schinke, Timur Alexander Yorgan

**Affiliations:** 1 Department of Osteology and Biomechanics, University Medical Center Hamburg-Eppendorf, 20246 Hamburg, Germany; 2 PolyGene AG, 8153 Rümlang, Switzerland; 3 ETH Phenomics Center (EPIC), ETH Zürich, 8092 Zürich, Switzerland; 4 Neuronal and Cellular Signal Transduction, Center for Molecular Neurobiology Hamburg (ZMNH), University Medical Center Hamburg-Eppendorf, 20246 Hamburg, Germany; 5 Center for Molecular Neurobiology Hamburg (ZMNH), University Medical Center, Hamburg-Eppendorf, 20246 Hamburg, Germany; 6 Institute for Translational Immunology and Research Center for Immunotherapy, University Medical Center, Johannes Gutenberg University, D 55131 Mainz, Germany; 7 Faculty of Medicine and University Hospital Cologne, Department of Pediatrics, University of Cologne, 50937 Cologne, Germany

**Keywords:** Calcium and phosphate metabolic disorders, Bone quality and biomechanics

## Abstract

The recent identification of homozygous WNT1 mutations in individuals with osteogenesis imperfecta type XV (OI-XV) has suggested that WNT1 is a key ligand promoting the differentiation and function of bone-forming osteoblasts. Although such an influence was supported by subsequent studies, a mouse model of OI-XV remained to be established. Therefore, we introduced a previously identified disease-causing mutation (*G177C*) into the murine *Wnt1* gene. Homozygous *Wnt1*^*G177C/G177C*^ mice were viable and did not display defects in brain development, but the majority of 24-week-old *Wnt1*^G177C/G177C^ mice had skeletal fractures. This increased bone fragility was not fully explained by reduced bone mass but also by impaired bone matrix quality. Importantly, the homozygous presence of the *G177C* mutation did not interfere with the osteoanabolic influence of either parathyroid hormone injection or activating mutation of LRP5, the latter mimicking the effect of sclerostin neutralization. Finally, transcriptomic analyses revealed that short-term administration of WNT1 to osteogenic cells induced not only the expression of canonical WNT signaling targets but also the expression of genes encoding extracellular matrix modifiers. Taken together, our data demonstrate that regulating bone matrix quality is a primary function of WNT1. They further suggest that individuals with WNT1 mutations should profit from existing osteoanabolic therapies.

## Introduction

Osteoporosis, the most prevalent skeletal remodeling disorder, is primarily treated by antiresorptive drugs inhibiting the differentiation and/or activity of osteoclasts.^1^ Although osteoanabolic treatment options, stimulating osteoblast-mediated bone formation, are available, they are not commonly used to treat osteoporotic patients.^2^ In the case of teriparatide, a fragment of parathyroid hormone (PTH), this is mostly explained by the relatively high costs and the mode of application (daily injection). In the case of romosozumab, an antibody blocking the action of the bone formation inhibitor sclerostin, there is still no long-lasting clinical experience, as this type of treatment was approved only recently.^3^ This explains why it is of highest clinical relevance to uncover additional molecular mechanisms regulating bone formation and skeletal integrity to optimize the treatment of not only osteoporosis but also various other skeletal disorders.

Combined research efforts in recent decades have revealed that the WNT signaling pathway is a key regulator of bone metabolism.^4^ This research area was initially triggered by the genetic analysis of individuals with sclerosing bone disorders, which are characterized by increased osteoblast activity. In these patients, either activating mutations of the putative WNT coreceptor LRP5 or inactivating mutations of the secreted protein sclerostin were identified as disease-causing.^5,6^ It was subsequently found that sclerostin binds to LRP5 to antagonize WNT signaling and thereby bone formation and that high bone mass mutations of LRP5 abolish the interaction with sclerostin.^7,8^ Because it is still unknown which of the 19 known WNT ligands physiologically promotes osteoblast activity in interaction with LRP5, it was a highly relevant observation that mutations of the *WNT1* gene were identified in patients with skeletal disorders.^9–12^ More specifically, whereas a subset of individuals with early-onset osteoporosis (EOOP) carry heterozygous *WNT1* mutations, homozygous *WNT1* mutations were found to be associated with osteogenesis imperfecta type XV (OI-XV).

These findings were initially surprising, as WNT1, based on a severe and lethal phenotype of *Wnt1*-deficient mice, was considered to be an essential factor for the development of the central nervous system.^13,14^ Consistent with human genetic findings, however, *swaying* mice harboring a spontaneous mutation in the *Wnt1* gene display severe osteoporosis, in addition to the initially identified neuronal phenotype.^15^ Consistently, osteoporosis with associated fractures was also reported for mice with specific *Wnt1* deletion in osteoblast linage cells, and we recently showed that inducible *Wnt1* expression in osteoblasts of transgenic mice causes a rapid bone mass increase.^16–18^ Finally, we established a mouse line harboring the EOOP-causing mutation R235W within the *Wnt1* gene, where we observed a low bone mass phenotype but no skeletal fractures.^19^

In the present manuscript, we describe the generation and analysis of a mouse model in which we introduced an OI-XV-causing mutation (*G177C*) into the *Wnt1* gene. Based on a previously published in silico analysis, this mutation is likely to destabilize the core of the WNT1 ligand and to impair its activity as a stimulator of canonical Wnt signaling.^11^ We observed that *Wnt1*^*G177C/G177C*^ mice, similar to the respective patient, did not display a brain pathology but had decreased bone mass and skeletal fractures. We additionally utilized this model to demonstrate that the homozygous presence of the *G177C* mutation does not diminish the osteoanabolic response toward PTH injection or LRP5 activation, and we further identified that impaired bone matrix quality in *Wnt1*^*G177C/G177C*^ mice contributes to skeletal fragility.

## Results

### Effects of homozygous *WNT1* mutations in patients

**Fig. 1:**
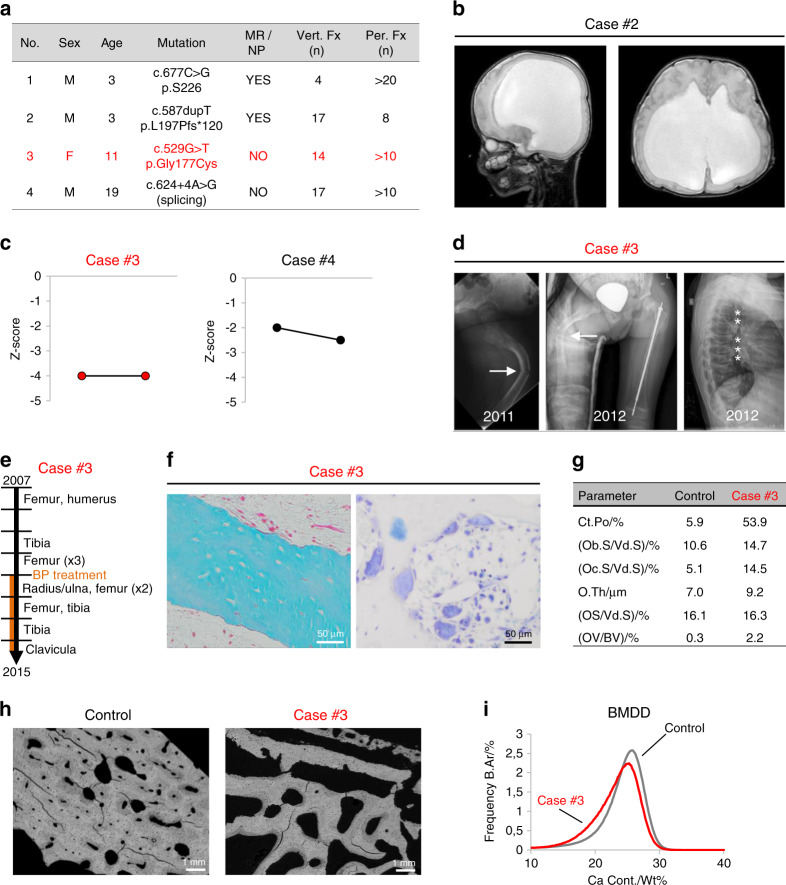
Characteristics of OI patients with homozygous WNT1 mutations. **a** Overview of four OI-XV patients, including sex, age, WNT1 mutation, presence of mental retardation/neurological phenotype (MR/NP), and number of vertebral and peripheral fractures (Vert. Fx, Per. Fx). **b** Cranial magnetic resonance imaging (cMRI, sagittal and axial view) showing hydrocephalus and brain atrophy in patient #2. **c** Dual X-ray energy absorptiometry (DXA) Z-scores in the two patients without brain effects at baseline and after 1 year (case #3) or 3 years (case #4) of neridronate treatment. **d** Radiographs of patient #3 at the age of 5 years receiving neridronate treatment for one year showing a left diaphyseal femur fracture (arrow) before (left) and after (middle) surgical treatment via telescopic nail. Note the new right diaphyseal femur fracture (arrow). Spine radiography of the same patient revealed multiple vertebral fractures indicated by asterisks (right). **e** Timeline of fractures that occurred in patient #3. The orange line indicates the period of neridronate treatment. **f** Histological images of a proximal femur biopsy (cortical bone) from case #3 indicating an absence of osteomalacia (left, Trichrome-Goldner staining) and several multinucleated osteoclasts (right, toluidine blue staining). **g** Histomorphometric quantification of cortical porosity (Ct. Po), osteoblast surface per void surface (Ob.S./Vd.S), osteoclast surface per void surface (Oc.S./Vd.S), osteoid thickness (O.Th.), osteoid surface (OS/Vd.S) and osteoid volume (OV/BV) measured in the cortical region of a proximal femur biopsy from case #3 in comparison to a location-, age- and sex-matched control biopsy. **h** Quantitative backscattered electron imaging (qBEI) comparing the cortical region of the bone biopsy from Case #3 to a control biopsy. Gray levels indicate the mineral content. **i** Bone mineral density distribution (BMDD) histograms identify a slight shift toward lower mineralization in the patient biopsy (case #3)

We identified four OI-XV patients with homozygous *WNT1* mutations, and not all of them displayed neurological symptoms (Fig. 1a). More specifically, while two 3-year-old children with *WNT1* mutations displayed a severe neurological phenotype with brain atrophy (Fig. 1b), two other children, aged 11 and 19 years, only had skeletal affection, and no further neurological diagnostics were initiated for these patients. A second observation was that bisphosphonate treatment, which had been initiated in all patients, did not lead to increased bone mineral density (Fig. 1c) and did not prevent the occurrence of long bone deformity or additional fractures in the presented patient cohort (Fig. 1d, e).

We also obtained a bone biopsy of the proximal femoral metaphysis from one of the patients during orthopedic surgery (Fig. 1f). In comparison to data obtained from an age- and sex-matched control biopsy, we observed an apparent imbalance of bone remodeling, i.e., slightly higher osteoblast numbers and more strongly activated osteoclastogenesis (Fig. 1g). However, the lack of established reference ranges for these skeletal sites prevents a precise evaluation of these parameters. We further applied quantitative backscattered electron imaging (qBEI) to determine the distribution of calcium within the mineralized bone matrix (Fig. 1h). Here, we found a shifted bone mineral density distribution (BMDD), i.e., decreased calcium content in the patient´s biopsy, when compared to an age- and sex-matched control (Fig. 1i). Taken together, these data show that homozygous *WNT1* mutations can negatively affect skeletal remodeling and bone matrix quality without causing neurological symptoms. Moreover, while other forms of OI are amenable to bisphosphonate therapy,^20^ this treatment option has limited efficacy in OI-XV patients.

### *Wnt1*^*G177C/G177C*^ mice display a bone-specific phenotype

**Fig. 2:**
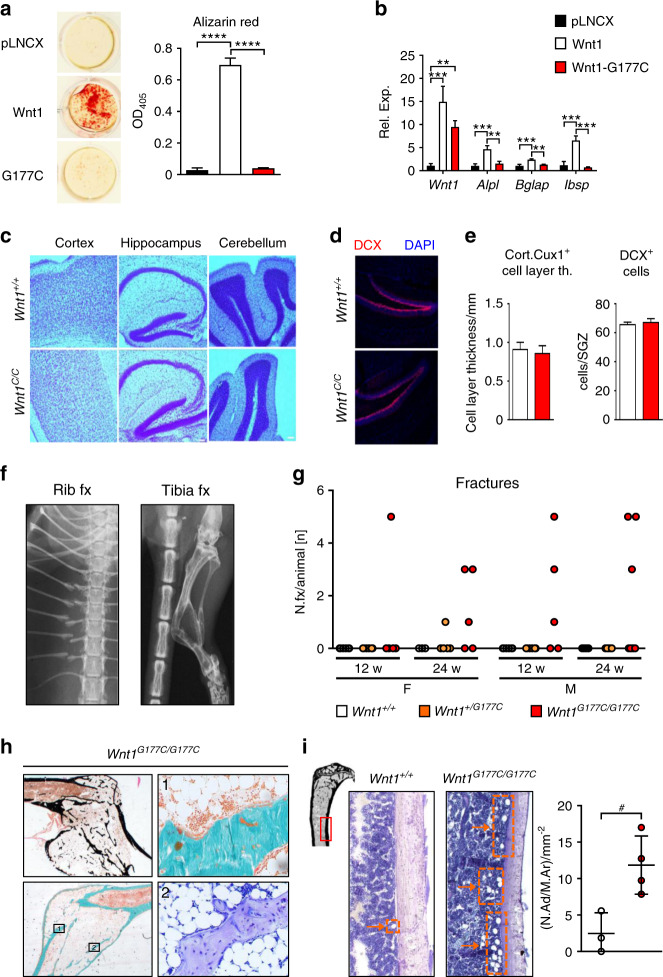
*Wnt1*^*G177C/G177C*^ mice display a bone-specific phenotype. **a** Representative images and quantification of alizarin red staining of ST2 cells transfected with empty vector (pLNCX) and expression plasmids for wild-type (*Wnt1*) or mutant *WNT1*(*G177C*) after 15 days of osteogenic differentiation. *n* = 3 samples per group. **b** Expression analysis by qRT–PCR of the indicated genes in ST2 cells transiently transfected with the same plasmids 5 days after transfection. *n* = 3 samples per group. **c** Representative Nissl staining of cortex, hippocampus, and cerebellum sections from 4-week-old *Wnt1*^*+/+*^ and *Wnt1*^*G177C/G177C*^ mice. **d** Representative immunofluorescent images of the hippocampus in the same mice. Red: Anti-doublecortin (DCX); Blue: DAPI. **e** Quantification of the cortical Cux1-positive cell layer thickness and of the doublecortin-positive neuroblasts in the subgranular zone of the hippocampus. *n* = 3 animals per genotype. **f** Representative contact radiographs of spontaneous fractures in 24-week-old *Wnt1*^*G177C/G177C*^ mice. **g** Number of spontaneous fractures observed per animal in female and male *Wnt1*^*+/+*^, *Wnt1*^*+/G177C*^, and *Wnt1*^*G177C/G177C*^ mice at 12 and 24 weeks of age. Each symbol represents an individual mouse. **h** Histological images of a tibial fracture callus from a 24-week-old *Wnt1*^*G177C/G177C*^ mouse. Top left: von Kossa/van Gieson stain; bottom left: Masson-Goldner stain, squares indicate areas magnified on the right side as indicated by the numbers. Top right: Masson-Goldner stain; bottom right: toluidine blue stain. **i** Representative images of tibial bone marrow from nonfractured 24-week-old wild-type or *Wnt1*^*G177C/G177C*^ mice and quantification of the number of bone marrow adipocytes per marrow area (N.Ad/M.Ar). The red rectangle on the symbolic tibia in the top left corner indicates the region depicted in the representative images. Arrows and boxes indicate regions with high numbers of adipocytes. Data were analyzed by one-way ANOVA with Dunnett’s multiple comparison test (**a**, **b**) or Student’s *t*-test (**e**, **i**). *P < 0.05, **P < 0.01, ***P < 0.001, ****P < 0.000 1. Error bars indicate standard deviation

To develop an appropriate mouse model of OI-XV, we intended to introduce the *G177C* mutation (identified in case 3) into the murine *Wnt1* gene. In the first step, we analyzed the influence of this mutation on osteogenic differentiation of the mesenchymal cell line ST2.^19^ We, therefore, introduced the *G177C* mutation into a *Wnt1* expression plasmid that was used for stable transfection. When we performed alizarin red staining 15 days after the addition of osteogenic medium, we observed that the positive influence of WNT1 was fully abrogated by the introduced mutation (Fig. 2a). Moreover, to investigate the more immediate influences of *Wnt1* overexpression, we performed qRT–PCR on ST2 cells osteogenically differentiated for 5 days after transient transfection. This revealed that the expression of osteoblast differentiation markers (*Alpl, Bglap, Ibsp*) was significantly induced by the wild-type but not by the mutant *Wnt1* expression plasmid (Fig. 2b).

This led us to introduce the *G177C* mutation into the murine *Wnt1* locus to generate a mouse model of OI-XV (Supplementary Fig. 1a). *Wnt1*^*+/+*^*, Wnt1*^*G177C/+*^, and *Wnt1*^*G177C/G177C*^ mice were born at the expected Mendelian ratio, and the latter did not display postnatal lethality or obvious abnormalities (Supplementary Fig. 1b). Importantly, in contrast to *Wnt1*^*sw/sw*^ or *Wnt1*-deficient mice,^13,14,21^ *Wnt1*^*G177C/G177C*^ mice did not display histological anomalies in different brain regions at 4 weeks of age (Fig. 2c). In fact, no morphological anomalies were detected in the cortex, hippocampus or cerebellum of these mice. Furthermore, the layer thickness of cortical Cux1-positive cells and the number of doublecortin (DCX)-positive neuroblasts in the subgranular zone were unaltered (Fig. 2d, e). We further followed phenotype progression in *Wnt1*^*G177C/G177C*^ mice over time and observed, in the absence of obvious extraskeletal defects, a high rate of spontaneous fractures. More specifically, as assessed by contact X-rays, the majority of 24-week-old *Wnt1*^*G177C/G177C*^ animals, but not their *Wnt1*^*+/+*^ littermates, displayed skeletal fractures, mostly in the rib cage and in the tibiae (Fig. 2f, g). This was confirmed by undecalcified histology, where we additionally observed an overabundance of adipocytes in the fracture callus (Fig. 2h). Of note, when we quantified the number of adipocytes per marrow area in nonfractured tibiae, there was no difference in the metaphysis (5.8 ± 2.9 adipocytes/mm² in wild-type vs. 4.2 ± 1.2 in *Wnt1*^*G177C/G177C*^ mice), but we observed significantly increased adipocyte numbers in the diaphysis of 24-week-old *Wnt1*^*G177C/G177C*^ mice (Fig. 2i). Collectively, these data underscored the adequacy of the *Wnt1*^*G177C/G177C*^ mice as a model of OI-XV.

### Trabecular bone mass is only moderately affected in *Wnt1*^*G177C/G177C*^ mice

**Fig. 3:**
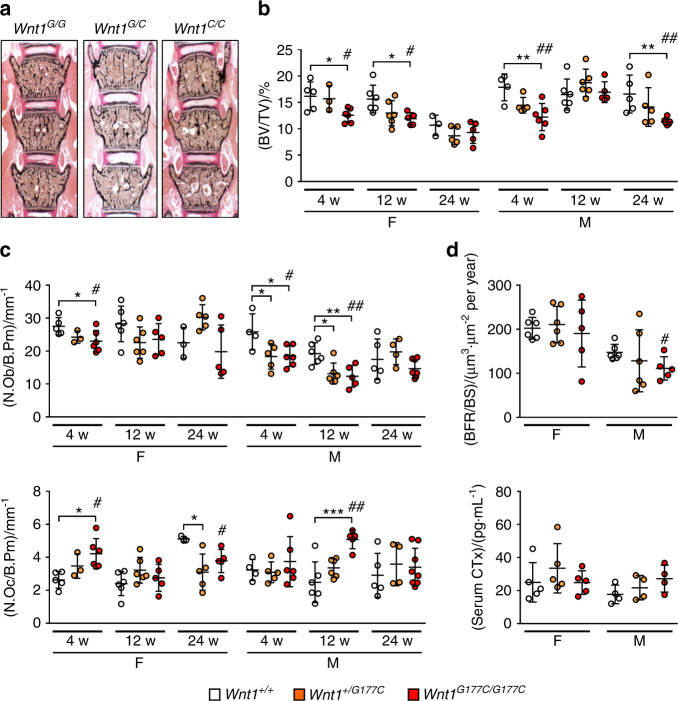
Trabecular bone mass is only moderately affected in *Wnt1*^*G177C/G177C*^ mice. **a** Representative undecalcified spine sections from 4-week-old male mice with the indicated genotypes (*Wnt1*^*G/G*^ = *Wnt1*^*+/+*^, *Wnt1*^*G/C*^ = *Wnt1*^*+/G177C*^ and *Wnt1*^*C/C*^ = *Wnt1*^*G177C/G177C*^) after von Kossa/van Gieson staining. **b** Quantification of the trabecular bone volume (BV/TV, bone volume per tissue volume) in female and male *Wnt1*^*+/+*^, *Wnt1*^*+/G177C*^, and *Wnt1*^*G177C/G177C*^ mice at 4, 12, and 24 weeks of age. **c** Quantification of the osteoblast (N.Ob/B.Pm, number of osteoblasts per bone perimeter) and osteoclast numbers (N.Oc/B.Pm, number of osteoclasts per bone perimeter) in the same groups of mice. **d** Quantification of the bone formation rate (BFR/BS, bone formation rate per bone surface) in the trabecular compartment of the lumbar spine (top) and serum crosslaps (CTx) levels (bottom) of male and female *Wnt1*^*+/+*^, *Wnt1*^*+/G177C*^ and *Wnt1*^*G177C/G177C*^ mice at the age of 12 weeks. Data were analyzed by one-way ANOVA with Dunnett’s multiple comparison test (*P < 0.05, **P < 0.01, ***P < 0.001) and Student’s *t*-test (*Wnt1*^*G177C/G177C*^ vs. *Wnt1*^*+/+*^, ^#^*P* < 0.05, ^##^*P* < 0.01). Error bars indicate standard deviation

To characterize the skeletal phenotype of *Wnt1*^*G177C/G177C*^ mice in greater detail, we first analyzed undecalcified spine sections (Fig. 3a). Here, we studied *Wnt1*^*+/+*^*, Wnt1*^*G177C/+*^, and *Wnt1*^*G177C/G177C*^ littermates of both sexes at the ages of 4, 12, and 24 weeks. When we quantified the trabecular bone volume per tissue volume (BV/TV), we observed a significant decrease in *Wnt1*^*G177C/G177C*^ mice of both sexes at the age of 4 weeks compared to either wild-type or heterozygous littermates (Fig. 3b). The extent of this trabecular osteopenia, however, did not increase with age, and there were only significant differences between the groups in 12-week-old female or 24-week-old male mice. When we quantified the osteoblast number per bone perimeter (N.Ob/B.Pm) we made a similar observation, i.e., a significant reduction in *Wnt1*^*G177C/G177C*^ mice of both genders only at 4 weeks of age (Fig. 3c). At that age, there was also a significant increase in the osteoclast number per bone perimeter (N.Oc/B.Pm) in female *Wnt1*^*G177C/G177C*^ animals, but overall, the histomorphometric changes in the trabecular bone compartment were unexpectedly moderate. Similarly, when we quantified bone cell activities at 12 weeks of age, we observed a significant reduction in the bone formation rate per bone surface (BFR/BS) only in male *Wnt1*^*G177C/G177C*^ mice but no change in the serum concentrations of the bone resorption biomarker CTx (Fig. 3d). Although these data are in principal agreement with the previously established role of *Wnt1* in skeletal remodeling, they did not provide a reasonable explanation for the high fracture incidence associated with the WNT1^*G177C*^ mutation.

### The WNT1^*G177C*^ mutation primarily affects the quantity and quality of cortical bone

**Fig. 4:**
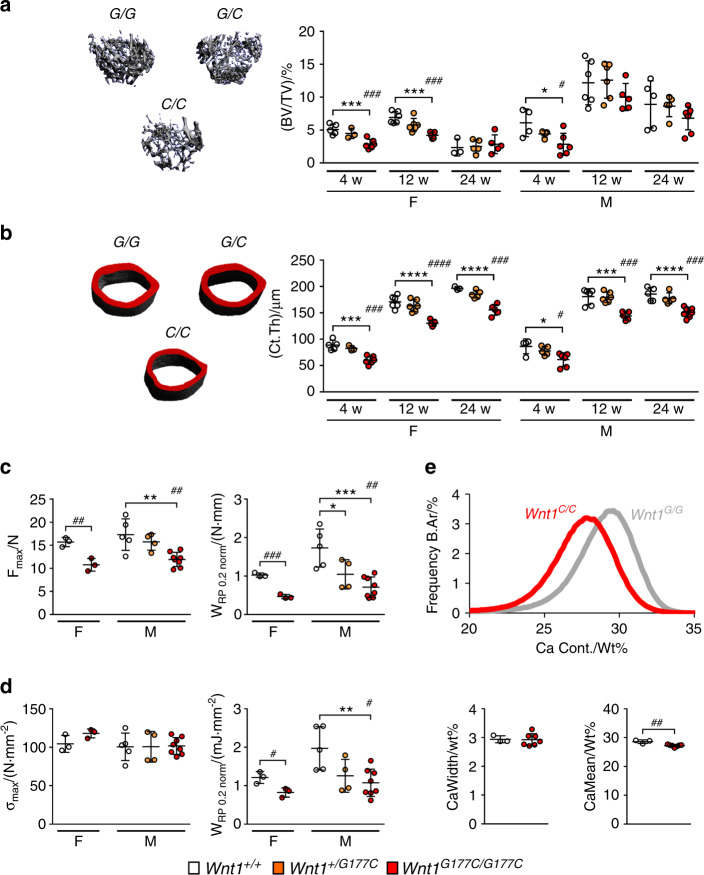
The WNT1^*G177C*^ mutation primarily affects the quantity and quality of cortical bone. **a** Representative µCT images of the trabecular bone in the distal femoral metaphysis from 12-week-old female mice. Quantification of the trabecular bone volume in female and male *Wnt1*^*+/+*^ (*G/G*), *Wnt1*^*+/G177C*^ (*G/C*), and *Wnt1*^*G177C/G177C*^ (*C/C*) mice at 4, 12, and 24 weeks of age is given on the right. **b** Representative µCT images of the cortical bone in the central femoral diaphysis from 12-week-old female mice. Quantification of the cortical thickness in female and male *Wnt1*^+/+^ (*G/G*), *Wnt1*^*+/G177C*^ (*G/C*), and *Wnt1*^*G177C/G177C*^ (*C/C*) mice at 4, 12, and 24 weeks of age is given on the right. **c** Quantification of mechanical properties of the femora from 24-week-old female and male mice with the indicated genotypes as determined by 3-point bending (F_max_: Maximum force that the bone could withstand; W_Rp0.2_: Work required to induce plastic deformation). **d** Quantification of the same parameters after normalization for morphological differences. **e** qBEI-based analysis of calcium distribution (top) and quantification of the calcium content (bottom) in the cortical compartment of tibiae from 24-week-old male wild-type and *Wnt1*^*G177C/G177C*^ mice. Data were analyzed by one-way ANOVA with Dunnett’s multiple comparison test (*P < 0.05, **P < 0.01, ***P < 0.001, ****P < 0.000 1) and Student’s *t*-test (*Wnt1*^*G177C/G177C*^ vs. *Wnt1*^*+/+*^, ^#^*P* < 0.05, ^##^*P* < 0.01, ^###^*P* < 0.001, ^####^*P* < 0.000 1). Error bars indicate standard deviation

We additionally analyzed the femora from the same groups of mice by µCT scanning. Here, we found that the heterozygous or homozygous presence of the WNT1^*G177C*^ mutation had no influence on the femur length of any age group (Supplementary Fig. 2a). Consistently, we did not detect an alteration of the chondrogenic growth plates in tibia sections of 4-week-old *Wnt1*^*G177C/G177C*^ mice (Supplementary Fig. 2b). Importantly, when we analyzed the trabecular bone compartment in the different groups of animals, we again found only a moderate negative impact of the WNT1^*G177C*^ mutation, in full agreement with the spine histology described above (Fig. 4a). In contrast, the cortical thickness in the femoral midshaft was significantly reduced in male and female *Wnt1*^*G177C/G177C*^ mice of all age groups, thereby demonstrating that the WNT1^*G177C*^ mutation primarily impacts the cortical bone compartment (Fig. 4b). The differential impact of the WNT1^*G177C*^ mutation on trabecular and cortical bone raised the question of whether WNT1 is specifically produced in cortical bone osteoblasts. We addressed this question by immunohistochemistry, but we did not detect differences between osteoblasts from the different bone compartments (Supplementary Fig. 3), in full agreement with a published transcriptome analysis.^22^ Moreover, as we previously observed that *Pls3*-deficient mice display similarly reduced cortical thickness without fractures,^23^ it remains to be addressed whether the rather moderate bone mass reduction observed in adult *Wnt1*^*G177C/G177C*^ mice can fully explain their skeletal fragility.

To analyze the potential impact of the WNT1^*G177C*^ mutation on bone quality, we, therefore, performed three-point bending assays of femora from *Wnt1*^*+/+*^ and *Wnt1*^*G177C/G177C*^ mice. Here, we observed that the latter displayed reduced biomechanical resistance to deformation (Fig. 4c), as expected. Importantly, however, when normalizing for the differences in bone morphology, the maximum resistive stress (σ_max_) of *Wnt1*^*G177C/G177C*^ femora was not changed toward that of wild-type littermates, but the work required to induce plastic deformation (W_Rp0.2_) was still significantly reduced (Fig. 4d). W_Rp0.2_ is mainly driven by the mineral content of the tissue; thus, we determined the mineralization status of the cortical bone matrix in *Wnt1*^*+/+*^ and *Wnt1*^*G177C/G177C*^ mice by further applying quantitative backscattered electron imaging. We thereby observed an overall shift toward lower mineralization, together with significantly reduced calcium content of the mineralized bone matrix (Fig. 4e), similar to the observed BMDD shift in the patient. These data indicate that the WNT1^*G177C*^ mutation affects not only the quantity but also the quality of the cortical bone matrix.

### The WNT1^*G177C*^ mutation does not interfere with the osteoanabolic influence of PTH injection or LRP5 activation

Because the skeletal status of OI-XV patients was not positively influenced by bisphosphonate treatment, we took advantage of *Wnt1*^*G177C/G177C*^ mice to study their response to osteoanabolic treatment options. We first addressed the question of whether the WNT1^*G177C*^ mutation would impair the responsiveness of osteoblasts to PTH, as the thereby mediated stimulation of bone formation has been linked to the activation of WNT signaling.^24,25^ We first cultured wild-type and *Wnt1*^*G177C/G177C*^ primary bone marrow cells, where we administered PTH for 6 h after 10 days of ex vivo osteogenic differentiation. Although matrix mineralization was moderately reduced in *Wnt1*^*G177C/G177C*^ cultures (Supplementary Fig. 4a), the PTH-responsive genes *Cited1* and *Tnsfsf11* (ref. ^26^) were induced to the same extent as in wild-type cultures (Supplementary Fig. 4b). Consistently, treatment of wild-type and *Wnt1*^*G177C/G177C*^ littermate mice by daily injection of PTH for two weeks resulted in increased periosteal bone formation and elevated serum levels of the bone formation biomarker PINP, regardless of the genotype (Supplementary Fig. 4c). These data demonstrate that the *G177C* mutation of WNT1 does not prevent the osteoanabolic response to intermittent PTH injections.

**Fig. 5:**
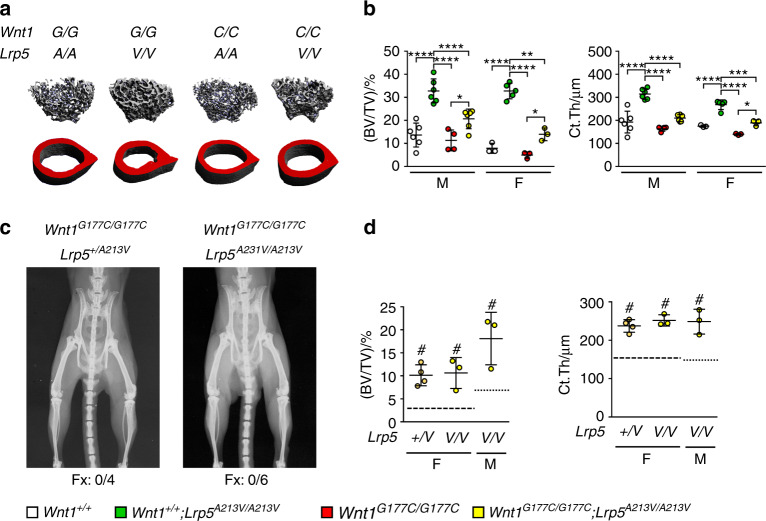
Assessment of osteoanabolic treatment options in *Wnt1*^*G177C/G177C*^ mice. **a** Representative µCT images of the distal femoral trabecular (top) and cortical (bottom) bone of 12-week-old wild-type (*+/+*;*+/+*), *Lrp5*^*A213V/A213V*^ (*+/+*;*A/A*), *Wnt1*^*G177C/G177C*^ (*G/G*;*+/+*) and *Lrp5*^*A213V/A213V*^*/Wnt1*^*G177C/G177C*^ (*G/G*;*A/A*) mice. **b** Quantification of the trabecular bone volume and cortical thickness in male and female mice. Data were analyzed by two-way ANOVA with Sidak’s multiple comparison test (*P < 0.05, **P < 0.01, ***P < 0.001, ****P < 0.000 1). **c** Representative contact radiographs of 24-week-old *Wnt1*^*G177C/G177C*^ mice carrying one or two *Lrp5*^*A213V*^ alleles. The number below the radiographs indicates the number of animals with fractures (Fx) per total number of animals in the group. **d** Quantification of the trabecular bone volume and cortical thickness in the femur of 24-week-old *Wnt1*^*G177C/G177C*^ mice of the indicated sex and *Lrp5* genotype. The dashed lines indicate the respective values in *Wnt1*^*G177C/G177C*^ mice with two wild-type *Lrp5* alleles. Data were analyzed by Student’s *t*-test (mutant *Lrp5* alleles vs. two wild-type *Lrp5* alleles, ^#^*P* < 0.05). Error bars indicate standard deviation

Because children are usually not treated with teriparatide, it was important to analyze the potential responsiveness of *Wnt1*^*G177C/G177C*^ mice to another type of osteoanabolic treatment option, i.e., antibody-mediated blockade of sclerostin. For that purpose, we took advantage of *Lrp5*^*A213V/A213V*^ mice, because the presence of the *A213V* mutation prevents the binding of sclerostin to LRP5 and because we have previously demonstrated that these mice are resistant to the influence of increased sclerostin expression.^27^ Thus, the *Lrp5*^*A213V/A213V*^ mice partially mimicked the effect of antibody-mediated sclerostin neutralization. We generated mice carrying sole or combined mutations of *Wnt1* and *Lrp5* and studied their skeletal phenotype at 12 weeks of age. By µCT scanning of femoral bones, we found that both trabecular bone volume and cortical thickness were significantly higher in *Wnt1*^*G177C/G177C*^*; Lrp5*^*A213V/A/213V*^ littermates than in *Wnt1*^*G177C/G177C*^*; Lrp5*^*+/+*^ littermates (Fig. 5a and b). Moreover, at 24 weeks of age, the heterozygous or homozygous presence of the *Lrp5*^*A213V*^ allele fully prevented the occurrence of skeletal fractures in *Wnt1*^*G177C/G177C*^ mice (Fig. 5c) and again resulted in increased trabecular and cortical bone mass (Fig. 5d). Taken together, these observations demonstrate that an *OI-XV* mutation of *Wnt1* does not prevent the osteoanabolic influence of LRP5 activation, which is an important finding in terms of patient treatment.

### WNT1 induces a specific transcriptional response indicating canonical WNT signaling activation

**Fig. 6:**
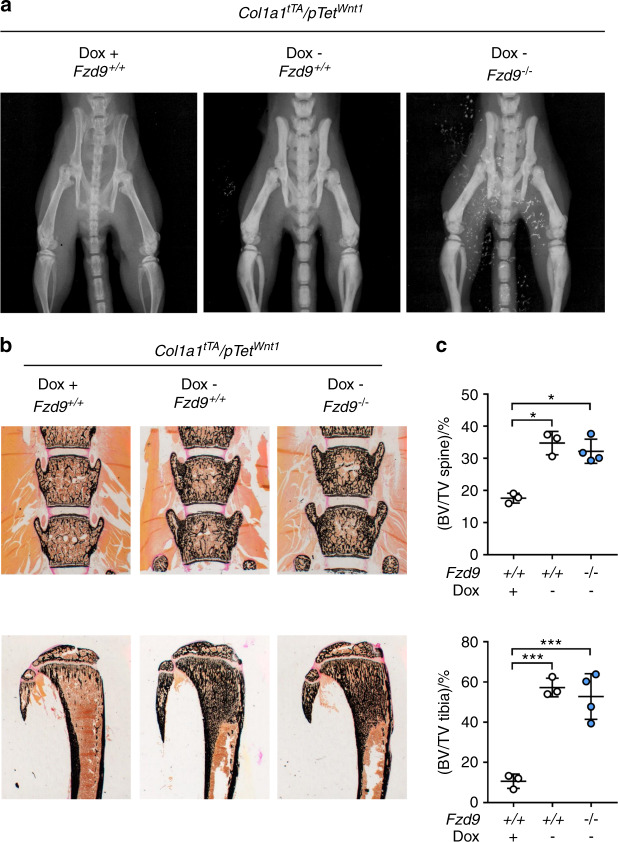
FZD9 is dispensable for the osteoanabolic action of *Wnt1*. **a** Representative contact X-rays of 6-week-old female *Col1a1*^*tTA*^*/pTet*^*Wnt1*^*/Fzd9*^*+/+*^ or *Col1a1*^*tTA*^*/pTet*^*Wnt1*^*/Fzd9*^*−/−*^ mice, in which *Wnt1* expression was silenced (Dox+) or activated by doxycycline removal for 1 week (Dox−). **b** Representative undecalcified spine and tibia sections from the same mice after von Kossa/van Gieson staining. **c** Trabecular bone mass, as determined by undecalcified histology of the spine and tibia. Data were analyzed by two-way ANOVA with Sidak’s multiple comparison test (*P < 0.05, **P < 0.01, ***P < 0.001). Error bars indicate standard deviation

The unaffected response of *Wnt1*^*G177C/G177C*^ mice toward LRP5 activation also supported our previous finding obtained in transgenic mice (*Col1a1*^*tTA*^/*pTet*^*Wnt1*^) with inducible osteoblast-specific *Wnt1* expression.^18^ Here, we demonstrated that the osteoanabolic influence of WNT1 was not abrogated by LRP5 deficiency.^18^ In the present study, we performed a similar experiment to address the question of whether FZD9 is the main receptor that mediates the osteoanabolic influence of *Wnt1* overexpression. In our opinion, the identification of such a receptor is highly relevant, and we considered FZD9 an excellent candidate, as we have previously reported that deletion of *Fzd9* in mice impairs bone formation.^28^ Therefore, we generated *Col1a1*^*tTA*^/*pTet*^*Wnt1*^ mice additionally lacking FZD9, in which we induced *Wnt1* expression by doxycycline removal at the age of 5 weeks. After one week of *Wnt1* induction, we observed, by contact X-ray (Fig. 6a), undecalcified spine histology (Fig. 6b) and subsequent histomorphometric quantification (Fig. 6c), the same bone mass increase in *Col1a1*^*tTA*^/*pTet*^*Wnt1*^/*Fzd9*^*−*/*−*^ mice as we did in *Col1a1*^*tTA*^/*pTet*^*Wnt1*^/*Fzd9*^*+/+*^ littermates.

**Fig. 7:**
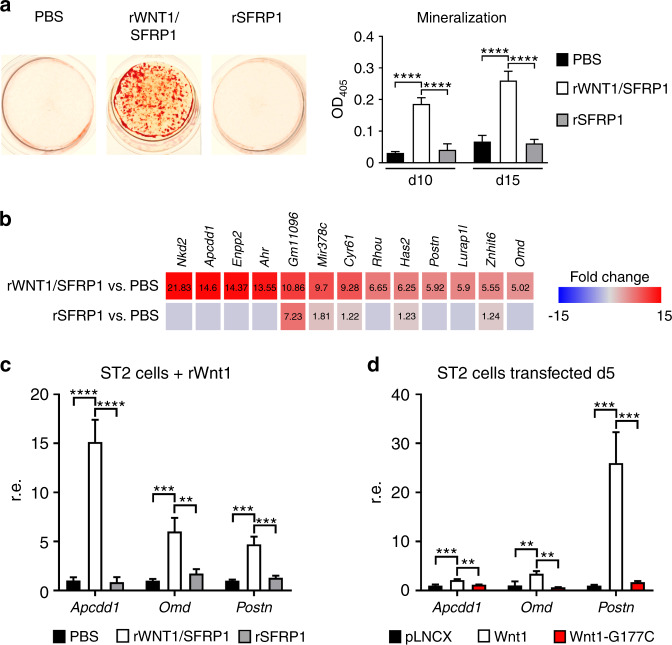
Intact WNT1 induces a specific transcriptional response indicating canonical Wnt signaling activation. **a** Representative images of alizarin red staining of ST2 cells continually treated with recombinant murine WNT1/SFRP1 complex, SFRP1 alone or PBS after 15 days of osteogenic differentiation. The quantification of the mineralization at Day 10 and Day 15 of differentiation is given on the right. Data were analyzed by one-way ANOVA with Tukey’s multiple comparison test (****P < 0.000 1). *n* = 3 samples per group. **b** Genome-wide expression analysis of ST2 cells after 6 h of stimulation with the WNT1/SFRP1 complex, SFRP1 alone or PBS. Shown are all genes displaying a more than five-fold induction by the WNT1/SFRP1 complex compared to PBS-treated cells. The numbers in the boxes indicate a positive fold-change. The second line shows the effect of SFRP1 alone. **c** qRT–PCR-based verification of the observed induction of *Apcdd1, Omd* and *Postn*. **d** Expression of the same genes in ST2 cells transiently transfected with either empty vector (pLNCX) or with expression plasmids for wild-type (*Wnt1*) or mutant *Wnt1*(*G177C*) 5 days after transfection. Data were analyzed by one-way ANOVA with Tukey’s multiple comparison test (**P < 0.01, ***P < 0.001, ****P < 0.000 1). *n* = 3 samples per group. Error bars indicate standard deviation

Because we have previously shown that FZD9 does not significantly influence the canonical WNT signaling pathway in osteoblasts, we next analyzed the influence of WNT1 on ST2 cells using an unbiased approach. We hereby aimed to identify potential downstream effectors and specific components that facilitate WNT1 signal transduction and potentially explain the phenotype of *Wnt1*^*G177C/G177C*^ mice. Here, we took advantage of a commercially available recombinant WNT1/SFRP1 protein complex, which was added to the medium of cultured ST2 cells in comparison to SFRP1 alone. We first confirmed that long-term treatment with WNT1/SFRP1 promoted the osteogenic differentiation of ST2 cells, as assessed by alizarin red staining (Fig. 7a). This led us to compare the transcriptomes of ST2 cells after short-term administration of WNT1/SFRP1 or SFRP1 for 6 h. Here, we identified four known target genes of canonical Wnt signaling, i.e., *Nkd2, Apccd1, Ahr*, and *Cyr61* (refs. ^29–32^), that were among the 13 genes displaying a more than 5-fold induction after WNT1/SFRP1 administration (Fig. 7b). Moreover, whereas the expression of established osteoblast differentiation markers was only moderately affected (Supplementary Fig. 5), there were two genes encoding extracellular matrix modifiers, i.e., *Postn* and *Omd*, which were strongly induced by WNT1/SFRP1.

After confirming this short-term induction by qRT–PCR (Fig. 7c), we additionally analyzed whether the WNT1^*G177C*^ mutation interferes with this transcriptional activation. Here, we took advantage of ST2 cells that were stably transfected with wild-type and mutant *Wnt1* expression plasmids. We thereby not only confirmed the WNT1-dependent transcriptional activation of *Apcdd1, Postn*, and *Omd* but also demonstrated that this induction was fully blunted by the WNT1^*G177C*^ mutation (Fig. 7d). Remarkably, in this experimental setting, *Postn* was the most strongly induced gene among all tested candidates (see also Fig. 2b). We were also able to detect a significant decrease in osteomodulin content in bone extracts isolated from *Wnt1*^*G177C/G177C*^ mice in comparison to WT controls (Supplementary Fig. 6). Taken together, these findings demonstrate that WNT1 modulates the expression of specific genes in osteoblast progenitor cells, indicating a canonical WNT signaling response and the control of at least one gene, i.e., *Postn*, encoding a matrix protein with known relevance for cortical bone integrity.^33^

## Discussion

Given the key role of the putative Wnt signaling regulators LRP5 and sclerostin in controlling osteoblast-mediated bone formation in mice and humans,^5–8^ it was highly relevant that several studies have identified *WNT1* mutations in individuals with either EOOP or OI-XV.^9–12^ In contrast to osteoporosis, which may have different causes and mostly affects adults, OI is a heterogeneous group of rare monogenetic disorders negatively affecting connective tissues but primarily the quality of the bone matrix in early childhood.^34^ The majority of OI forms are related to defective type I collagen fibrils as a consequence of mutations in either the type I collagen genes (*COL1A1, COL1A2*) or in genes required for posttranslational modification of type I collagen (*LEPRE1, PLOD2*, and others). In addition, some OI forms are not directly related to collagen processing but rather represent a state of severely impaired osteoblast differentiation or function.^35^ This latter group also includes OI-XV, which can alternatively be regarded as the most severe form of WNT1-dependent EOOP. Because the vast majority of OI patients are currently treated with bisphosphonates, aiming at reducing fracture risk,^20^ we also initiated such treatment for OI-XV patients, yet none of them responded in a satisfactory manner. This absence of treatment success was one of the major driving forces for us to generate a valid mouse model for OI-XV.

We chose to introduce the WNT1^*G177C*^ mutation because the respective patient did not display neurological symptoms. Consistently, *Wnt1*^*G177C/G177C*^ mice developed a pronounced bone phenotype, but none of the brain abnormalities previously described for *Wnt1*^*−/−*^ or *Wnt1*^*sw/sw*^ mice.^13,14,21^ Indeed, similar to our previously published mouse model of WNT1-dependent EOOP caused by the WNT1^*R235W*^ mutation,^19^ *Wnt1*^*G177C/G177C*^ mice displayed a bone-specific phenotype, despite the ubiquitous presence of the mutation. In contrast to *Wnt1*^*R235W/R235W*^ mice, however, the newly established *Wnt1*^*G177C/G177C*^ model displays not only a skeletal phenotype with earlier onset but also a high incidence of skeletal fractures. A similar fracture rate was previously described for either *Wnt1*^*sw/sw*^ mice, in addition to their cerebellum phenotype,^15^ or for *Wnt1*^*fl/fl;Runx2-Cre*^ and *Wnt1*^*fl/fl;Dmp1-Cre*^ mice, where WNT1 loss-of-function was restricted to osteoblast lineage cells.^16,18^ It is therefore important to conclude that *Wnt1*^*G177C/G177C*^ mice are the first animal model in which a germline *Wnt1* mutation causes skeletal fractures without affecting the central nervous system. As this also applies to the respective patient and because there are thus far no human *WNT1* mutations reported to exclusively affect brain development, it is reasonable to postulate that controlling skeletal integrity is the predominant physiological function of WNT1.

A second relevant finding of our study was that *Wnt1*^*G177C/G177C*^ mice display a high rate of skeletal fractures, although their bone mass reduction was unexpectedly moderate. In fact, we have previously analyzed other genetically modified mouse models with much stronger phenotypes, where we did not observe spontaneous fractures until the age of 24 weeks.^27,36^ It was also evident that the homozygous presence of the WNT1^*G177C*^ mutation primarily affected cortical bone. Changes in trabecular bone volume and cellular parameters were mostly consistent in 4-week-old mice but displayed some variance between other age groups and sexes. These fluctuations might be caused by the transition from the modeling to the remodeling phase of bone turnover as well as the onset of age-related bone loss that differs between male and female mice, possibly due to altered hormone and cytokine profiles.^37^ In contrast, cortical thickness was consistently reduced in male and female *Wnt1*^*G177C/G177C*^ mice of all age groups.

Structurally, the phenotype of *Wnt1*^*G177C/G177C*^ mice resembles the phenotype of *Wnt16*-deficient mice, an established regulator of cortical bone mass.^38^ Importantly, however, whereas the action of WNT16 was primarily attributed to an osteoprotegerin-mediated indirect influence on osteoclastogenesis, WNT1 was established as an osteoanabolic ligand, which is also reflected by our previous analysis of mice with inducible *Wnt1* overexpression.^18^ Moreover, a published RNA sequencing analysis revealed that the expression of *Wnt16*, but not of *Wnt1*, is much higher in cortical than in cancellous bone.^22^ In line with these results, we did not observe a difference in WNT1 immunoreactivity between trabecular or cortical bone by immunohistochemistry. Therefore, it remains to be addressed whether WNT1 and WNT16 act via similar downstream effectors or independently from each other. With regard to the high fracture incidence in *Wnt1*^*G177C/G177C*^ mice, it is also important to refer to our previous analysis of *Pls3*-deficient mice, a model of X-linked osteoporosis. Here, we observed a similar reduction in cortical bone mass as we did in *Wnt1*^*G177C/G177C*^ mice, yet *Pls3*-deficient animals did not display spontaneous fractures.^23,39^ This led us to hypothesize that the *G177C* mutation also affects bone quality, which was supported by qBEI indicating reduced mineral content of the *Wnt1*^*G177C/G177C*^ bone matrix. Likewise, as determined by three-point bending assays, we detected impaired mechanical properties of *Wnt1*^*G177C/G177C*^ femora, independent of the differences in bone morphology.

Having established *Wnt1*^*G177C/G177C*^ mice as a model for OI-XV, we utilized them to assess their response to existing osteoanabolic treatment options. In fact, as both PTH treatment and LRP5 activation could depend on intact WNT1 signaling, it was not necessarily expected that the *Wnt1*^*G177C/G177C*^ mice would react in the same way as wild-type controls. The observed normal response to osteoanabolic PTH in *Wnt1*^*G177C/G177C*^ is in line with results from *Wnt1*^R235W^ mice and with clinical data in few patients with WNT1-dependent EOOP.^18,19^ However, while such treatment might be a preferable option for adult patients with EOOP, there is a safety concern regarding teriparatide treatment in children, as animal studies have demonstrated an increased risk of osteosarcoma development.^25,40^ Therefore, it was important to assess whether the introduction of an LRP5 mutation, which disables the inhibitory action of sclerostin, into the *Wnt1*^*G177C/G177C*^ background would cause an osteoanabolic response, partially mimicking the effect of an anti-sclerostin antibody. Because both trabecular and cortical bone mass were increased by the *Lrp5*^*A213V/A213V*^ genotype to a similar extent in wild-type and *Wnt1*^*G177C/G177C*^ mice and because we did not observe skeletal fractures in 24-week-old *Wnt1*^*G177C/G177C*^*; Lrp5*^*A213V/+*^ or *Wnt1*^*G177C/G177C*^*; Lrp5*^*A213V/A/213V*^ mice, it is reasonable to speculate that antibodies neutralizing sclerostin can mediate a beneficial osteoanabolic effect in OI-XV patients, especially when taken into consideration that these will interfere not only with the binding of sclerostin to LRP5 but also with LRP6. More specifically, it has been established that sclerostin can inhibit WNT signaling by binding to both of these coreceptors.^8^ Moreover, LRP5 and LRP6 have been shown to provide a similar and partially redundant function in the context of WNT signaling.^41^ Therefore, it will be important to address in future studies whether the osteoanabolic effect of WNT1 is blunted in mice with LRP6 inactivation in specific bone cell populations.

Regardless of this conclusion, the results obtained in *Wnt1*^*G177C/G177C*^*; Lrp5*^*A213V/A/213V*^ mice are also relevant for understanding molecular interactions explaining the effects of WNT1 on osteoblast lineage cells. More specifically, our experiments show that the osteoanabolic influence of nonantagonized LRP5 does not depend on an intact WNT1 ligand. They thereby support our previous observation showing that the osteoanabolic influence of WNT1 in *Col1a1*^*tTA*^*/pTet*^*Wnt1*^ mice does not require the LRP5 protein.^18^ In other words, there is now dual in vivo evidence, which strongly suggests that LRP5 is not a relevant coreceptor for WNT1. Therefore, a receptor complex mediating WNT1 binding and signaling in osteoblast lineage cells remains to be identified. Based on our previous finding that mice lacking FZD9 display reduced bone formation,^28^ we addressed the question of whether FZD9 is required for the osteoanabolic action of WNT1. We again used the *Col1a1*^*tTA*^*/pTet*^*Wnt1*^ model, in which a one-week induction of *Wnt1* expression in osteoblasts led to an approximate doubling of the trabecular bone mass, also in combination with FZD9 deficiency. The clarity of our findings strongly suggests that FZD9 is not a relevant WNT1 receptor in osteoblast lineage cells, which is additionally supported by our previous molecular studies showing that *Fzd9*-deficient primary osteoblasts had a normal canonical Wnt signaling response after administration of WNT3a.^28^ Therefore, the identification of a relevant receptor mediating the osteoanabolic influence of WNT1 requires future research efforts, ideally by unbiased screening approaches.

In another attempt to understand the molecular mechanisms explaining the influence of WNT1 on bone formation and matrix quality, we took advantage of the mesenchymal cell line ST2, for which we and others have previously observed a strong induction of osteogenic differentiation after transfection of a *Wnt1* expression plasmid.^16,19^ Because we aimed to identify WNT1-regulated target genes, we took advantage of a recombinant WNT1/SFRP1 complex, which allowed us to analyze transcriptomic changes after short-term administration. We observed a specific response that was not mediated by SFRP1 alone and that was indicative of canonical WNT signaling activation. Most importantly, however, whereas the expression of molecular markers for osteoblast differentiation or of genes mutated in other types of OI were not affected by WNT1/SFRP1 administration, there was a striking induction of two genes encoding extracellular matrix modifiers, i.e., *Postn* and *Omd*. The strong WNT1-mediated induction of these genes was also confirmed in transfected ST2 cells, and here, the *Wnt1*^*G177C*^ mutation fully abrogated this response. Indeed, based on our experiments, it appears that the *Wnt1*^*G177C*^ mutant does not convey an effect in ST2 cells and behaves similarly to a null variant. However, this conclusion is not fully consistent with the in vivo findings, as the phenotype of *Wnt1*^G177C/G177C^ mice is less severe than that of *Wnt1*^−/−^ or *Wnt1*^*sw/sw*^ mice,^13–15^ especially with regard to neuronal aspects and viability. Although it remains to be studied to what extent reduced *Postn* expression contributes to the skeletal fragility of *Wnt1*^*G177C/G177C*^ mice, it is important to state that there is strong in vivo evidence for a key role of periostin in controlling cortical bone integrity.^42^ *Postn*-deficient mice display altered bone material properties, particularly in the cortical compartment, thereby causing an increased injury response to fatigue loading and even bone damage (i.e., microcracks) in the absence of experimental stimuli.^43^ In contrast, the potential role of osteomodulin in bone formation and matrix integrity is still unclear, especially since a mouse deficiency model has not yet been phenotypically analyzed. Interestingly, however, it was shown by direct interaction assays that osteomodulin has a positive influence on the formation of type I collagen fibrils.^44,45^

Taken together, although it is evident that uncovering the precise molecular mechanisms explaining the action of WNT1 in bone remodeling requires additional research, we truly believe that our manuscript is important in several regards. First, by establishing the first mouse model for OI-XV, which displays a bone-specific phenotype, we fully confirmed that controlling skeletal integrity is a key function of WNT1 in mice. Second, our deep phenotypic analysis demonstrates that the mutational inactivation of WNT1 primarily affects the cortical bone compartment, not only quantitatively but also qualitatively. Third, based on the data obtained in *Wnt1*^*G177C/G177C*^ mice, it is reasonable to speculate that the WNT1 mutation does not interfere with the two available osteoanabolic treatment options, which is important information for clinicians. Moreover, it is evident that the identification of a specific receptor mediating the influence of WNT1 on bone formation and matrix integrity might pave the way to develop additional osteoanabolic treatment options for various bone loss disorders, including osteoporosis.

## Materials and methods

### Patient analysis

We identified four patients with clinical features of severe OI and homozygous mutations of the *WNT1* gene verified by genetic testing (Cases #1–4). Patients were specifically referred to pediatric neurologists for continuative diagnostics in case of discrepancies from physiological mental and/or motor development. Whereas two patients showed only a skeletal phenotype (Cases #3 and #4), two patients displayed additional neurological symptoms, such as pronounced mental retardation (Cases #1 and #2). For one patient displaying a severe neurological phenotype at birth, cranial magnetic resonance imaging was performed within the first week of life to detect brain anomalies (Case #2). All patients were treated with bisphosphonate injections (2 mg·kg^−^^1^ neridronate every 3 months) due to multiple long bone and/or vertebral fractures,^46^ and the treatment effect was followed by dual X-ray energy absorptiometry. If necessary, surgical treatment of long bone fractures or severe deformity was performed by implantation of intramedullary nails. At the time of surgery, a bone biopsy was taken from the proximal femoral metaphysis of one patient, with the written informed consent of the legal representatives (Case #3). The human femoral biopsy (obtained at the age of 5 years) was compared to an age-matched healthy control obtained in the context of a previous study.^47^

### Cell culture

The mesenchymal cell line ST2 (obtained from DSMZ, Germany) was cultured in α-MEM (Merck KGaA, Germany) supplemented with 10% fetal calf serum (GE Health care, USA) and 100 U·mL^−1^ penicillin-streptomycin (Thermo Fisher Scientific, USA). To analyze the influence of WNT1 on these cells, we took advantage of the plasmid *pLNCX*-*Wnt1* (kindly provided by Dr. J. Kitajewksi). The *G177C* mutation was introduced by the QuikChange II system (Agilent Technologies, USA) according to the manufacturer´s instructions. The presence of the introduced mutation was verified by Sanger sequencing (Microsynth AG, Switzerland). Transfection of ST2 cells was performed with the Lipofectamine 2000 system (Thermo Fisher Scientific, USA). More specifically, we transfected cells with an empty vector (plasmid pLNCX), the *Wnt1* expression plasmid or the *Wnt1*^*G177C*^ expression plasmid for 24 h. Subsequently, the cells were treated with 300 U·mL^−1^ G418-BC (Merck KGaA, Germany) for 5 days to select for stably transfected cells. After replating the surviving cells, we induced osteogenic differentiation by adding 50 μg·mL^−1^ ascorbic acid and 10 mmol·L^−1^ ß-glycerophosphate to the medium. After 15 days of differentiation, alizarin red staining was performed and quantified as previously described.^28,48^ To analyze short-term influences on gene expression, we also performed transient transfection experiments where we isolated RNA for expression analysis 5 days after transfection. For the evaluation of immediate effects on gene expression, cells were serum-starved overnight and stimulated with commercially available murine recombinant WNT1/SFRP1 complex (100 ng·mL^−1^) (Bio-Techne Corp., USA) for 6 h after serum starvation overnight. As controls, equimolar amounts of recombinant SFRP1 (Bio-Techne Corp., USA) or the carrier (PBS) alone were used. Bone marrow cells were isolated from 12-week-old *Wnt1*^*+/+*^ and *Wnt1*^*G177C/G177C*^ mice and plated at a density of 5 × 10^4^ cells per ml in α-MEM medium (Sigma–Aldrich Corp., USA) supplemented with 10% (v/v) FCS (Thermo Fisher Scientific Inc., USA) and 100 U·mL^−1^ penicillin/streptomycin (Life Technologies, USA). For osteogenic differentiation, 50 μg·mL^−1^ ascorbic acid and 10 mmol·L^−1^ β-glycerophosphate (both Sigma–Aldrich Corp., USA) were added to the medium 3 days after plating. The medium was changed every 2 to 3 days. Alizarin red staining was performed with cultured osteoblasts 10 days after differentiation as previously described (32). To monitor responsiveness to PTH, cells were serum-starved for 18 h and then stimulated with 10 nmol·L^−1^ hPTH (1–34) (Bachem Holding AG, Switzerland) for 6 h.

### Expression analysis

RNA isolation was performed using the Nucleospin RNA II kit (Macherey-Nagel GmbH & Co. KG, Germany). The concentration of RNA was measured using a NanoDrop ND-1000 system (Thermo Fisher Scientific Inc., USA). RNA quality and integrity were assessed by the TapeStation 2200 system (Agilent Technologies Inc., USA). For qRT–PCR expression analysis, the RNA was reverse transcribed using the Verso cDNA Synthesis Kit (Thermo Fisher Scientific Inc., USA) according to the manufacturer’s instructions. Quantitative expression analysis was performed using the StepOnePlus system and predesigned TaqMan gene expression assays (Thermo Fisher Scientific Inc., USA): *Alpl* (Mm00475834_m1), *Apcdd1* (Mm01257559_m1), *Bglap* (Mm03413826_mH), *Ibsp* (Mm00492555_m1), *Omd* (Mm00449589_m1), *Postn* (Mm00450111_m1), and *Wnt1* (Mm01300555_g1). *Gapdh* expression was used as an internal control. Relative quantification was performed according to the ΔΔCT method. Whole transcriptome analysis was performed utilizing the Clariom D mouse system (Thermo Fisher Scientific Inc., USA) according to the manufacturer’s GeneChip™ WT PLUS reagent kit manual (document 703174, revision A.0). In brief, 100 ng of total RNA from pooled samples of three independent experiments was used for the synthesis of 2nd-cycle ss-cDNA. Five micrograms of fragmented and labeled cDNA was subsequently used for gene chip hybridization. After washing and staining with Affymetrix Fluidics Station 450, microarrays were scanned with the Affymetrix Gene Chip Scanner 7G. The signals were analyzed with Transcriptome Analysis Console software (TAC 4.0, Thermo Fisher Scientific Inc., USA) using default analysis settings (version 1) and Gene + Exon - SST-RMA as summarization.

### Animal generation

*Wnt1*^*G177C*^ mutant mice were generated by Polygene AG (Switzerland) from embryonic stem cells modified via homologous recombination with a targeting construct harboring the disease-associated *Wnt1* c.529G > T and silent *Wnt1* c.534G > A mutations and a FRT flanking neomycin selection cassette later removed by crossing with a Flp-deleter line (B6.SJL-Tg(ACTFLPe)9205Dym/J). Genotyping was performed using the primers 5′-GTACCTGGGAAGCTGATCTC-3′ and 5′-TAGTCGCAGGTGCAGGACTC-3′, detecting a 404 bp wild-type or 503 bp *Wnt1*^*G177C*^ allele. For Sanger sequencing (Seqlab Sequence Laboratories GmbH, Germany) of the mutation site, the primers 5′-CAGCGTTCATCTTCGCAATC-3′ and 5′-GCCTGCTAATCTCTTCTG-3′ were utilized. *Lrp5*^A213V^ mutant mice were obtained from the Jackson Laboratory (#012671). The *Lrp5* allele was genotyped using the primers 5′ AGTACTGGCTGGCACAGA-3′ and 5′-CAGGCTGCCCTTGCA GAT-3′. *Col1a1*^*tTA*^*/pTet*^*Wnt1*^*/Fzd9*^*−/−*^ mice (STOCK-Tg(tetO-*Wnt1*,-luc)TWNTLach-Tg(Col1a1-tTa)139Niss-*Fzd9*^tm1Uta^/Uke) were generated by crossing osteoblast-specific inducible *Wnt1Tg* mice^18^ with *Fzd9*-deficient mice.^28^ Unless indicated otherwise, mice from this line were kept under a doxycycline-containing diet (ssniff-Spezialdiäten GmbH, Germany).

### Animal husbandry

Mice were housed in a specific pathogen-free environment with a 12-h light/dark cycle, 45%–65% relative humidity, and 20–24 °C ambient temperature in open or individually ventilated cages with wood shavings bedding and nesting material in groups not surpassing six animals. Mice had *ad libitum* access to tap water and standard rodent chow (1328P, Altromin Spezialfutter GmbH & Co. KG, Germany). After initiation of treatment, the welfare of mice was assessed daily based on overall appearance and body weight.

### Osteoanabolic PTH treatment of mice

*Wnt1*^*G177C*^ mice were treated for 2 weeks up to the age of 12 weeks with 80 μg·kg^−1^ per day hPTH (1–34) (Bachem Holding AG, Switzerland) by intraperitoneal injection. Littermate mice were allocated randomly to treatment groups (coin flip), and endpoint measurements (µCT and histomorphometry) were performed in a blinded fashion. No unexpected or severe adverse events were observed.

### Radiographic analysis

All skeletons were analyzed by contact radiography (35 kV, 2 s) (Faxitron XRay Corp., USA) after fixation for overall skeletal integrity. For µCT analysis, the right femur of each mouse was fixed and placed into a radiotranslucent sample holder. Scanning and analysis were performed as previously described^27^ with a voxel resolution of 10 µm using a µCT 40 desktop cone-beam microCT (Scanco Medical, Switzerland) according to standard guidelines.^49^ Trabecular bone was analyzed in the distal metaphysis in a volume situated 2 500–500 μm proximal to the distal growth plate. Cortical bone was analyzed in a 1 000 μm long volume situated in the middle of the diaphysis. Cortical bone evaluation was performed with a threshold of 300, whereas for trabecular bone, a threshold of 250 was used. The length of the femora was determined by the number of slices containing the bone.

### Histology

For bone histology, skeletons were fixed in 3.7% PBS-buffered formaldehyde for 48 h before they were transferred into 80% ethanol. The lumbar vertebral bodies L1 to L4 and the left tibia of each mouse were dehydrated in ascending alcohol concentrations before they were embedded in methylmethacrylate. Sections of 4 μm thickness were cut in the sagittal plane using a Microtec rotation microtome (Tachno-Med GmbH, Germany). Sections were stained by von Kossa/van Gieson and toluidine blue staining procedures as described previously.^32^ Histomorphometry was carried out according to the guidelines of the American Society for Bone and Mineral Research^50^ using an OsteoMeasure system (Osteometrics Inc., USA). Growth plate and adipocyte measurements were performed using the OsteoMeasure system (Osteometrics Inc., USA). For brain histology, the animals were sacrificed, and the brains were removed and immersed in 4% formaldehyde solution overnight at 4 °C. The brains were washed with deionized water, and then free-floating vibratome slices were rehydrated in descending grades of ethanol (100%, 90%, and then 70%). Afterward, the slices were stained with 0.5 % cresyl violet for 4 min, and the brain slices were immersed in water containing 5 drops of absolute glacial acetic acid for 30 s. Finally, the sections were dehydrated in ascending grades of 70% ethanol, 90% ethanol, and then 100% ethanol for fifteen minutes. The brain sections were then cleared in xylene for five minutes twice. Finally, all brain sections were mounted with Entellan® medium (Merck KGaA, Germany).

### Immunological assays

For immunohistochemical analysis of the brain, polyclonal rabbit anti-CUX1 (1:100, Cat# ABE217, Merck KGaA, Darmstadt, Germany) or polyclonal rabbit anti-doublecortin (anti-DCX) (1:400; Cat#326003, Synaptic Systems GmbH, Germany) antibodies were utilized. Nuclei were visualized by DAPI staining prior to mounting using Fluoromount (Merck KGaA, Germany). Images were taken using a Keyence Fluorescence Microscope (BZ-9000, Keyence Corp., Osaka, Japan) and processed with ImageJ software.^51^ For immunohistochemical detection of WNT1, tibiae from wild-type mice were dissected and immersed in 4% PFA in PBS, pH 7.2, overnight. Thereafter, they were decalcified in 10% EDTA for two weeks. Sixty-micrometer-thick vibratome sections were prepared and washed in PBS several times. Fluorescence immunohistochemistry was performed, and nonspecific binding sites were blocked for 30–60 min at RT (0.3% BSA, 10% horse serum, 0.3% Triton X-100 in PBS). The tissue was subsequently incubated overnight at 4 °C with primary anti-Wnt1 (GTX83320, GeneTex, Inc. USA) antibody 1:500 in carrier (0.2% BSA, 1% horse serum, 0.3% Triton X-100 in PBS). After several washes in PBS, sections were incubated for 1–2 h with Alexa488-coupled secondary antibody (Molecular Probes, Inc., USA) in the carrier, washed in PBS and mounted in ProLong Gold (Invitrogen, USA). Images were taken with an F1000 confocal microscope (Olympus Corp., Japan). Serum concentrations of procollagen I N-terminal peptide (PINP) (Cloud Clone Corp., USA) were measured using an antibody-based detection kit according to the manufacturer’s instructions.

Western blot detection of osteomodulin was performed on bone extracts from murine femoral diaphysis cortices. Samples were pulverized in liquid nitrogen, and matrix proteins were extracted by initial incubation in 4 mol·L^−1^ guanidin, 50 mmol·L^−1^ Tris/HCl for 48 h and a second incubation in 4 mol·L^−1^ guanidin, 0.5 mol·L^−1^ EDTA, 50 mmol·L^−1^ Tris/HCl buffer for additional 48 h. The supernatant of the second incubation step was dialyzed against H_2_O and concentrated using centrifuge membrane concentrators. Proteins were separated on 12% Bis-Tris gels by SDS–PAGE, and silver staining was used to determine overall loading. Osteomodulin was detected with an AF3308 antibody (1:2 000, Biotechne, USA), and P0449 was used as a secondary antibody (1:1 000, Agilent Technologies, Inc., USA). Luminescence signals were recorded using a digital detection hood (Bio–Rad Laboratories, Inc., USA).

### Bone mineral density distribution

For analysis of the BMDD, quantitative backscattered electron imaging (qBEI) was performed as described previously^52,53^ Embedded, carbon-coated mouse tibiae (and controls) as well as the human femoral biopsy from the patient (case #3) and a control individual^47^ were imaged using a scanning electron microscope (LEO 435 VP; LEO Electron Microscopy Ltd., UK) operated at 20 kV and 680 pA using a constant working distance of 20 mm (BSE Detector, Type 202; K.E. Developments Ltd., UK). The BMDD of the cross-sectioned bone was determined by the generated gray values using a custom MATLAB-based program (TheMathWorks, Inc., Natick, MA, USA).

### Biomechanical testing

For biomechanical testing, a three-point bending test was performed on dissected femora using a universal testing machine Z2.5/TN1S and testXpert software (both Zwick Roell, Germany). Femora were horizontally mounted and centrally positioned with the anterior surface facing downward at a bearing distance of 7 mm. A perforce of 2 N was applied to ensure no movement of the sample. A constant displacement rate of 0.05 mm·s^−1^ was applied. Load-displacement data were recorded at 100 Hz until failure. The results from the µCT analysis of the mid-diaphyseal area were utilized for normalization.

### Statistics

Data were analyzed by a two-tailed Student’s *t*-test using Excel software (Microsoft Corp., USA) when two genotypes were compared. For three groups, one-way ANOVA with Dunnett’s or Tukey’s multiple comparison test was applied as indicated (GraphPad Software Inc., USA). When comparing four groups with different alleles of two independent genetic loci, two-way ANOVA with Sidak’s multiple comparison test was used (GraphPad Software Inc., USA). Observed vs. expected frequencies were compared by *χ*² test using Prism software (GraphPad Software Inc., USA). All data are reported as the mean ± SD with additional points representing individual animals. A value of *P* ≤ 0.05 was considered statistically significant. All data have been provided within the manuscript.

### Study approval

All animal experiments were performed according to the Cantonal Veterinary of the Canton of Zurich, Swiss Federal Animal Welfare Law TSchG, Art. 11.1, Animal Welfare Regulations, Art. 142, Animal Experimentation Regulations, Art 9 and Annex1 or in accordance with the local implementation of EU Directive 2010/63/EU for animal experiments approved by the Ethics Committee of the University Medical Center Hamburg-Eppendorf and by the “Behörde für Soziales, Familie, Gesundheit und Verbraucherschutz” (G13/151, G14/039, G16/117, N073/2018, N053/2019, Org869). All patient data were obtained in accordance with the Declaration of Helsinki protocols and were approved by the local institutional review boards.

## Supplementary information

Supplementary Figures
