# Supplementary material for: The WNT1G177C mutation specifically affects skeletal integrity in a mouse model of osteogenesis imperfecta type XV
Source: Bone Res. 2021 Nov 10;9:48. doi: 10.1038/s41413-021-00170-0 (PMC8580994; doi:10.1038/s41413-021-00170-0)
Supplement: Supplementary file 1 — Supplementary Figures [file 41413_2021_170_MOESM1_ESM.pdf]

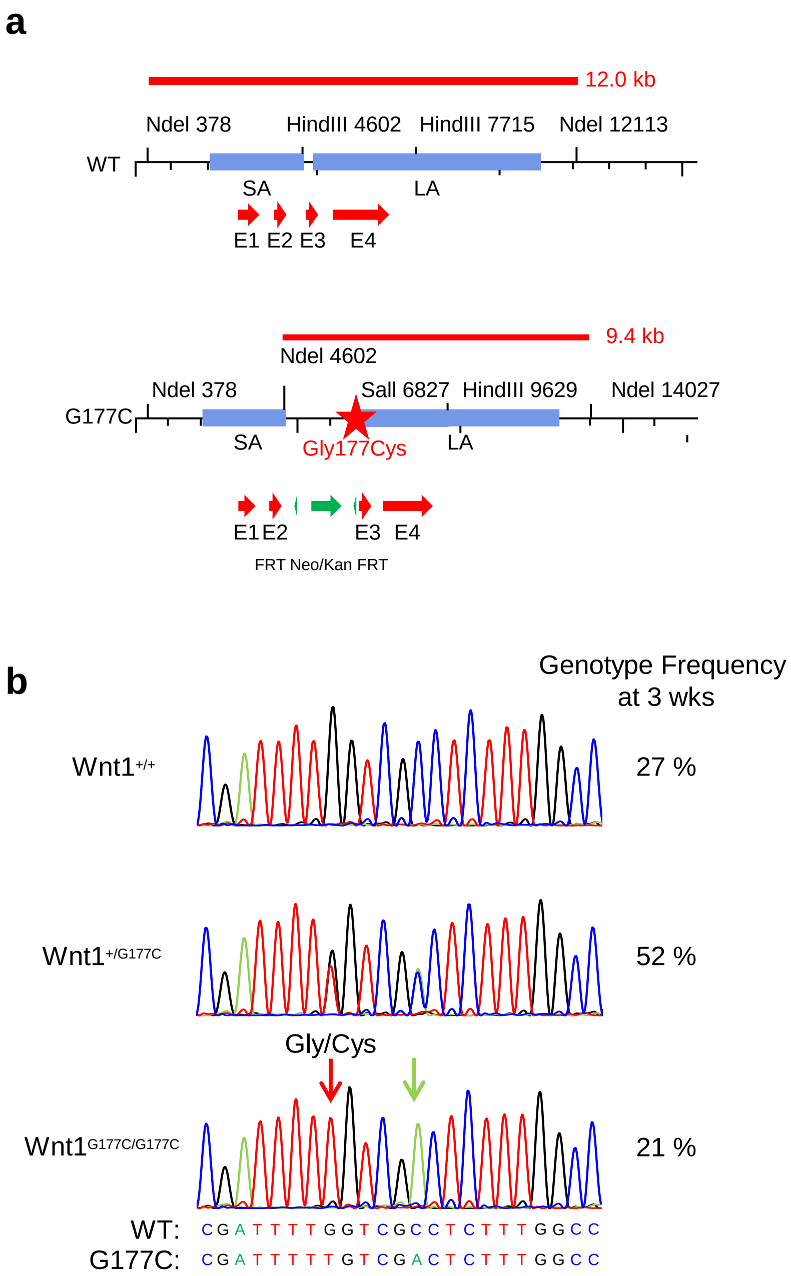


**Supplementary Figure 1.** Generation of *Wnt1*$^{G177C/G177C}$ mice. (**a**) Schematic presentation of the wildtype (WT) and modified (G177C) *Wnt1* allele before removal of the neomycin selection cassette. Thin red line: fragments detected by Southern blotting after restriction with NdeI. Blue bars: homologous regions used for recombination. SA: short arm, LA: long arm. Red star: introduced point mutation resulting in the G177C amino acid exchange. Red arrows: Wnt1 exons 1-4 as indicated. Green triangles: FRT sites. Green arrow: Neomycin resistance selection cassette. (**b**) Sanger sequencing results of the modified region of *Wnt1* in *Wnt1*$^{+/+}$, *Wnt1*$^{+/G177C}$ and *Wnt1*$^{G177C/G177C}$ mice. The red arrow indicates the mutation resulting in the amino acid exchange, the green arrow indicates a silent mutation. Numbers on the right indicate the genotype ratio of offspring at weaning from heterozygous matings. Observed frequencies were compared against expectation by $\chi^2$ test.

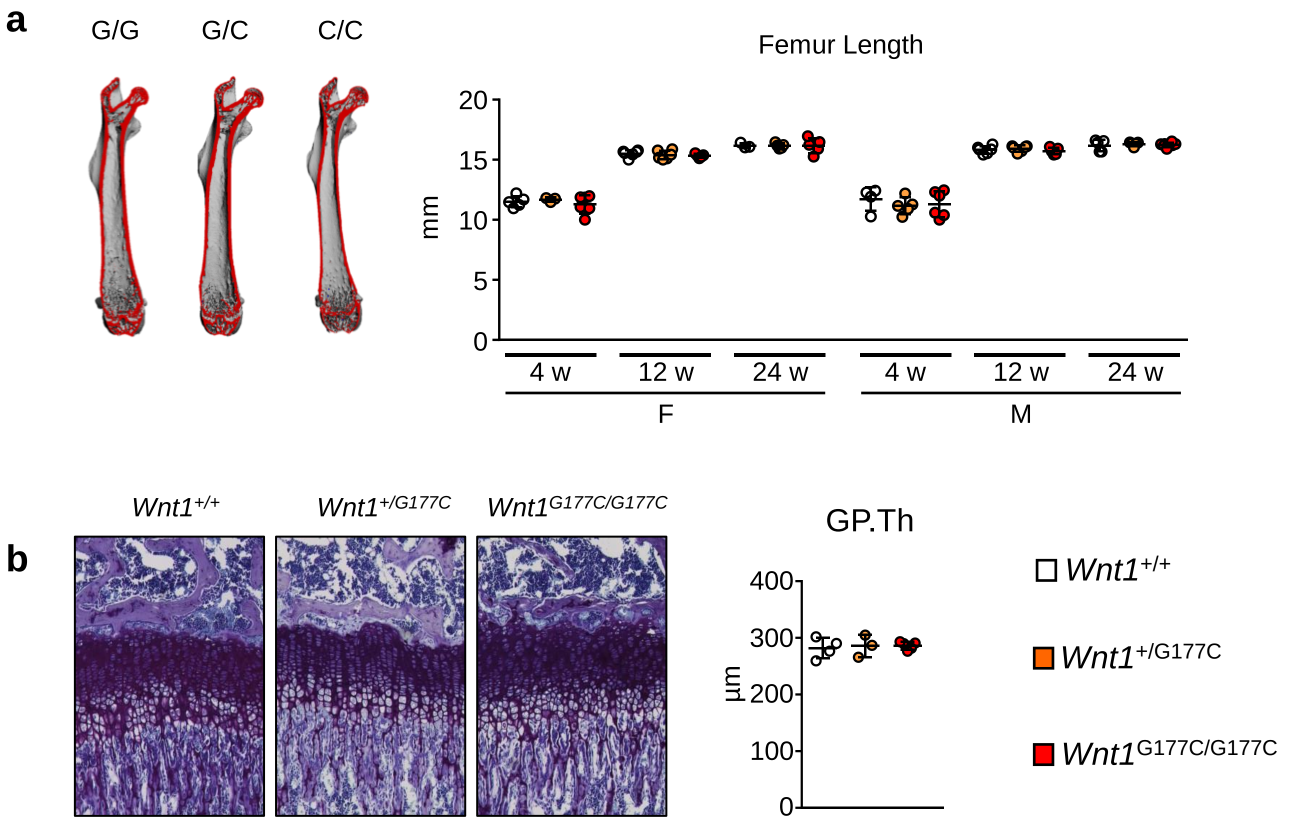


**Supplementary Figure 2.** Skeletal morphology *$Wnt1^{G177C/G177C}$* mice. (**a**) Representative µCT images of the femora from 12-week-old female mice. The virtual cutplane appears red. Quantification of the femur length of female and male *$Wnt1^{+/+}$* (G/G), *$Wnt1^{+/G177C}$* (G/C) and *$Wnt1^{G177C/G177C}$* (C/C) mice at 4, 12 and 24 weeks of age is given on the right. (**b**) Representative toluidine blue stained images and quantification of the whole growth plate thickness in the proximal femur of 4-week-old female mice with the indicated genotypes. Data were analyzed by a one-way ANOVA with Dunnett's multiple comparison test and Student's t-test (*$Wnt1^{G177C/G177C}$* vs. *$Wnt1^{+/+}$*). Error bars indicate standard deviation.

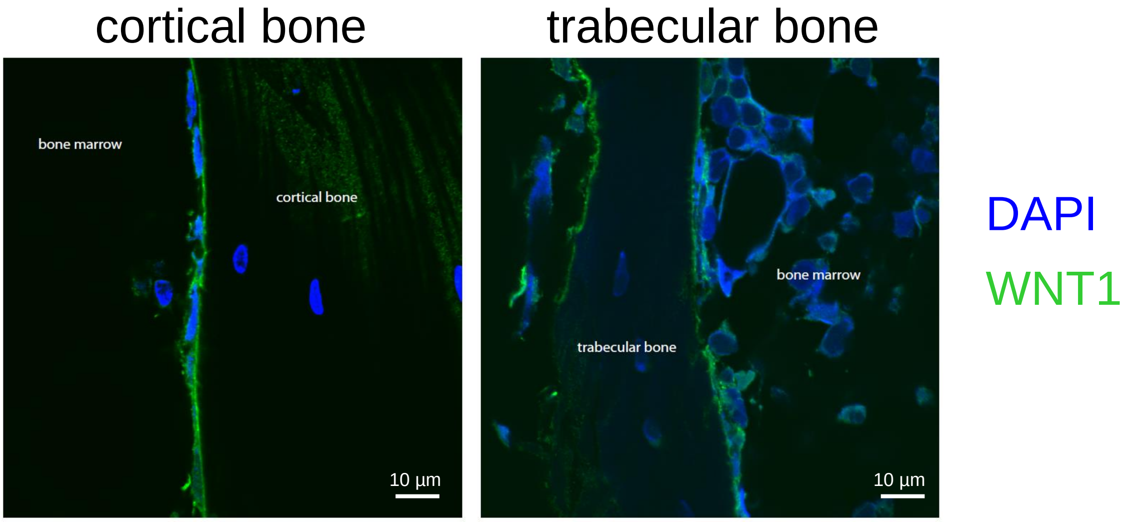


**Supplementary Figure 3.** Presence of WNT1 in cortical and trabecular bone. Representative immunohistochemical stainings showing WNT1 (green) and nuclei (DAPI, blue) in the cortical and trabecular compartment of a tibia from a female wildtype mouse.

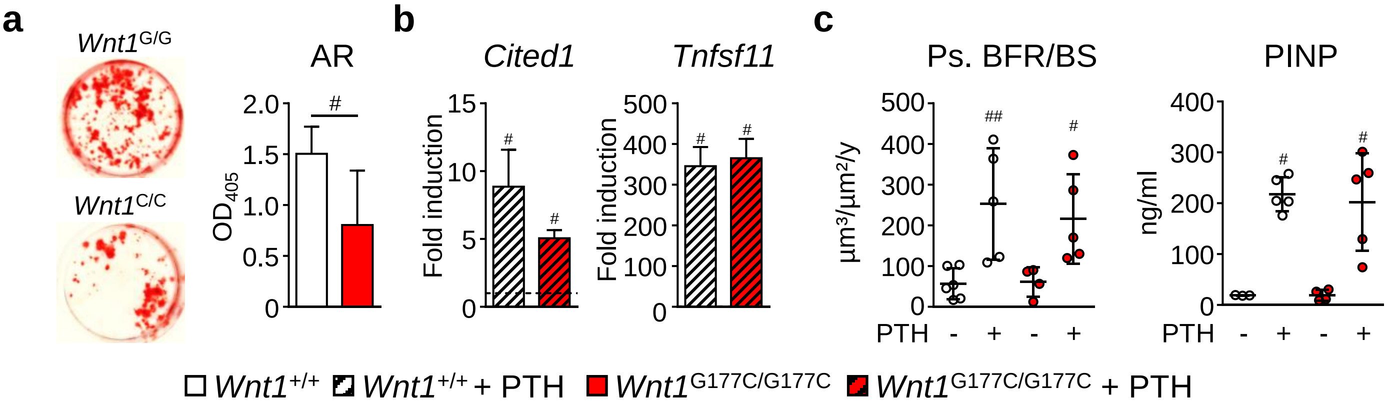


**Supplementary Figure 4.** PTH-response of *Wnt1^G177C/G177C^* mice. (**a**) Representative images of primary osteoblasts isolated from wildtype (*Wnt1^G/G^*) and *Wnt1^G177C/G177C^* (*Wnt1^C/C^*) mice after alizarin red staining. Quantification is given on the right. Data were analyzed by Student's t-test. #p<0.05, n = 3 independent experiments per genotype. (**b**) qRT-PCR expression analysis showing the fold-induction of the PTH-responsive genes *Cited1* and *Tnfsf11* after 6 h of treatment with 10 nM PTH. Data were analyzed by Student's t-test vs. control cultures of the same genotype represented by the dashed line. #p<0.05, n = 3 independent experiments per genotype. (**c**) Periosteal bone formation rate and serum PINP levels in 12-week-old wildtype or *Wnt1^G177C/G177C^* mice after two weeks of daily PTH injection. Data were analyzed by Student's t-test (treated vs. control animals of the same genotype, #p<0.05, ##p<0.01). Error bars indicate standard deviation.

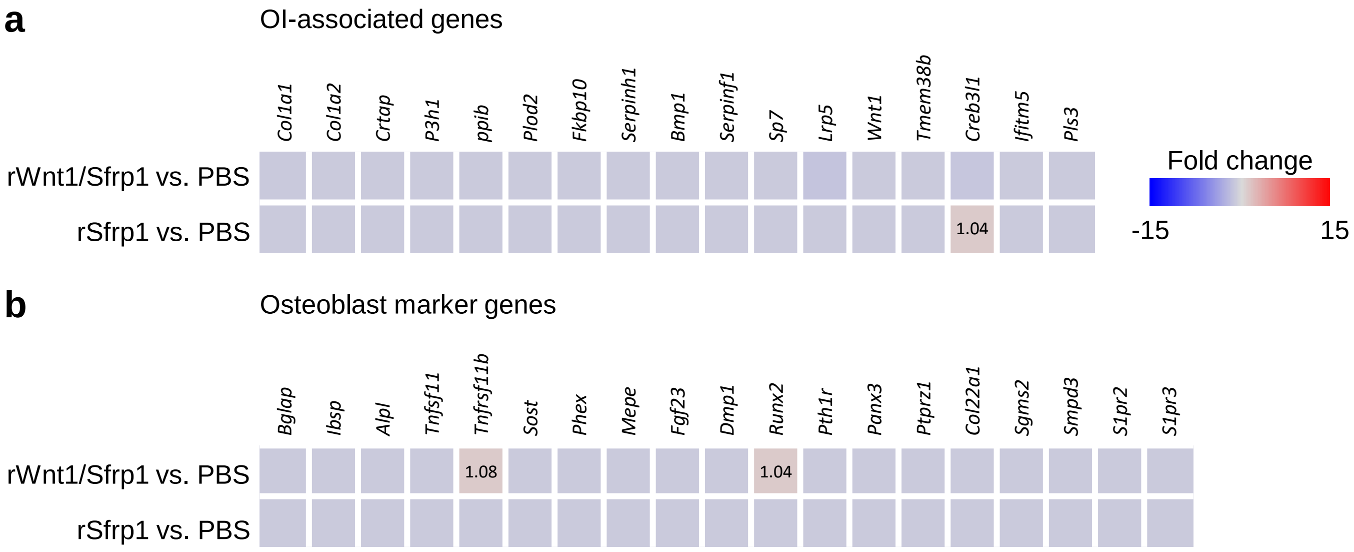


**Supplementary Figure 5.** WNT1 does not induce the expression of genes with relevance for bone integrity. Genome-wide expression analysis of ST2 cells after 6h of stimulation with the WNT1/SFRP1 complex, SFRP1 alone or PBS. Shown are (**a**) genes mutated in osteogenesis imperfecta or (**b**) encoding molecular markers of osteoblasts. The color of the boxes indicates the fold change as compared to PBS treated cells. The numbers in the boxes indicate a positive fold-change.

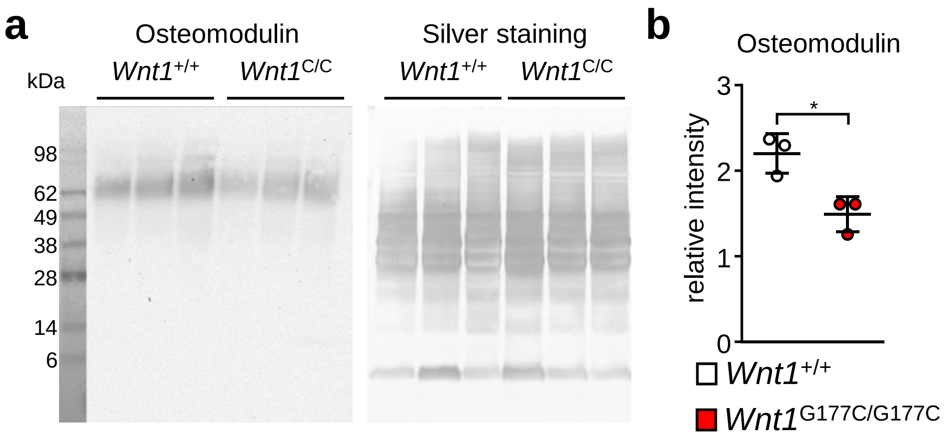


**Supplementary Figure 6.** Osteomodulin content of *Wnt1*$^{G177C/G177C}$ bones. a) Left: Western blot detecting Osteomodulin in matrix protein extracts from femoral cortical bone isolated from 6-week-old mice with the indicated genotypes. Right: Total protein silver staining as a loading control. b) Quantification of the Osteomodulin western blot signal intensity in relation to the average silver staining intensity of the respective samples. Data were analyzed by Student's t-test (*$p<0.05$). n = 3 samples per group. Error bars indicate standard deviation.
